# Unravelling head tremor mechanisms: insights from speech analysis in essential tremor and cervical dystonia

**DOI:** 10.1007/s00702-025-02965-5

**Published:** 2025-06-07

**Authors:** Jan Rusz, Petr Holly, Tereza Tykalova, Michal Simek, Tereza Hubena, Olga Ulmanova, Robert Jech, Radim Krupicka, Evzen Ruzicka

**Affiliations:** 1 Department of Circuit Theory, Faculty of Electrical Engineering, Czech Technical University in Prague, Technická 2, Prague, 160 00 Czechia; 2 Department of Neurology, Centre of Clinical Neuroscience, First Faculty of Medicine, Charles University and General University Hospital, Kateřinská 30, Prague, 120 00 Czechia; 3 Department of Biomedical Informatics, Czech Technical University in Prague, Kladno, Czech Republic

**Keywords:** Cervical dystonia, Essential tremor, Dysarthria, Speech disorder, Acoustic analysis

## Abstract

Head tremor is a common symptom in both essential tremor (ET) and cervical dystonia (CD). Distinguishing between these two conditions can be challenging in clinical practice, particularly when head tremor is the dominant feature. Our goal was to explore the potential of speech assessment in recognizing the mechanisms of head tremor in patients with ET and CD. Objective acoustic vocal assessments of oral diadochokinesis, phonatory stability, vocal tremor, and speech timing were performed. Of the 93 patients assessed, 39 had cervical dystonia (CD) with head tremor, 38 had ET with head tremor (ET-HT), and 16 had ET with no head tremor (ET-nHT). Compared to both CD and ET-nHT, ET-HT showed irregular sequential motion rate, excessive pitch fluctuations, increased noise, and higher extent of vocal vibrato. Compared to CD, ET-HT also demonstrated slower sequential motion rate, prolonged pauses, and a slower articulation rate. Additionally, ET-HT had more pronounced vocal tremolo compared to ET-nHT. Speech assessment provided discrimination between the CD and ET-HT groups with an area under curve of 0.80. This study underscores the promising potential of speech analysis in recognizing mechanisms of head tremor in patients with ET or CD, revealing more severe and distinct speech impairments in ET-HT patients compared to those with CD.

**Supplementary Information:**

The online version contains supplementary material available at 10.1007/s00702-025-02965-5.

## Introduction

Essential tremor (ET) is the most common movement disorder in adults. Currently, ET is diagnosed clinically based on the presence of bilateral upper-limb action tremor of at least three years duration (Bhatia et al. 2018). It may also involve tremors in other locations, such as the head, voice, and lower limbs, but must occur without other neurological signs like dystonia, ataxia, or parkinsonism (Bhatia et al. 2018). Head tremor is present in more than half of ET patients, more commonly in severe ET and in women (Louis 2025). However, diagnosis becomes challenging in patients with predominant head tremor, as dystonic tremor in cervical dystonia (CD) remains a possibility, even when dystonic posturing is questionable and upper limb tremor is present (Pandey et al. 2020). Furthermore, some patients with head tremor may develop CD years after the onset of their tremor (Ferrazzano et al. 2022; Rivest and Marsden 1990). Various clinical and neurophysiological tests have been proposed to assist in differentiating the background mechanisms of head tremor. For instance, it has been described that head tremor subsides in the supine position in patients with ET, whereas it persists in CD (Agnew et al. 2012). Further, a prolongation of the somatosensory temporal discrimination threshold has been reported in patients with tremor associated with dystonia and not in ET (Tinazzi et al. 2013). However, the usefulness of these tests has been questioned, and reliable and validated methods to distinguish essential and dystonic head tremor are still scarce (Holly et al. 2023).

One potential tool to aid in the differentiation between ET and CD with head tremor might be the analysis of speech abnormalities. It is known that ET is associated with vocal tremor and phonatory control issues (Gamboa et al. 1998; Hlavnicka et al. 2020; Suppa et al. 2021). Interestingly, the previous study even documented the triggering of head tremor following sustained phonation (Wright et al. 2014). Moreover, clinical, neuroimaging, and neuropathological studies have implicated the cerebellum in the pathogenesis of ET (Ma et al. 2012; Quattrone et al. 2008). Indeed, a former pilot study found that ET patients with additional clinical signs of cerebellar dysfunction but not ET patients with postural and/or simple kinetic tremor exhibited significantly increased speech timing abnormalities during syllable repetition (Kronenbuerger et al. 2009).

Therefore, we aimed to explore the diagnostic potential of speech assessment in distinguishing between ET and CD patients with head tremor. We hypothesized that ET patients with head tremor would manifest increased phonatory instability, vocal tremor, and speech timing abnormalities.

## Methods

### Study design

The study was approved by the Ethics Committee of the General University Hospital in Prague, Czech Republic, and has therefore been performed in accordance with the ethical standards established in the 1964 Declaration of Helsinki. All participants were duly informed about the objectives of the study and provided written informed consent.

From 2015 to 2021, research participants were recruited from the Movement Disorder Centre at the Department of Neurology, General University Hospital in Prague. The study invited patients diagnosed with ET, reclassified according to the current criteria (Bhatia et al. 2018), including ET with head tremor (ET-HT) and ET with no head tremor (ET-nHT). Additionally, patients with CD and head tremor were also recruited. Individuals with known genetic or secondary causes of dystonia, as well as those with comorbidities affecting neck position and movements, phonation, upper limb movements, stability, or gait, were excluded from the study. Healthy control subjects were recruited from the general community via advertisements. To be eligible for the study, they needed to be free of speech disorders, motor neurological disorders, active oncological illnesses, and psychoactive substance abuse.

### Clinical evaluation

For patients receiving botulinum toxin injections into neck muscles, a minimum interval of 12 weeks after their last dose was required. All patients were also instructed to discontinue their current tremor medications at least 24 h before the examination. Each patient underwent an evaluation by a neurologist (P.H.), who used a structured questionnaire to gather information on the family history of tremor, the patient’s symptoms and disease progression, comorbidities, and the effects of alcohol and medications. The clinical examination included the Essential Tremor Rating Assessment Scale (TETRAS) (Elble et al. 2012). Additionally, patients were evaluated using the Toronto Western Spasmodic Torticollis Rating Scale (TWSTRS) (Consky et al. 1990), and cerebellar function was assessed with the Scale for the Assessment and Rating of Ataxia (SARA) (Schmitz-Hubsch et al. 2006).

### Speech assessment

Speech recordings were performed in a quiet room with a low ambient noise level using a head-mounted condenser microphone (Beyerdynamic Opus 55, Heilbronn, Germany) placed approximately 5 cm from the subject’s mouth. Speech signals were sampled at 48 kHz with 16-bit resolution. All participants were instructed to perform two times (i) fast /pa/-/ta/-/ka/ syllable repetition for at least 10 times per 1 breath, (ii) sustained phonation of the vowel /a/ in one breath for as long and as steadily as possible, (iii) reading a short paragraph of standardized text composed of 80 words.

We performed an automated acoustic assessment of 8 representative speech features that were previously found to be sensitive to hyperkinetic dysarthria in general or specifically associated with essential tremor (Table 1) (Hlavnicka et al. 2020; Kouba et al. 2023; Rusz et al. 2021a). We evaluated oral diadochokinesis using a fast syllable repetition paradigm to detect *slow sequential motion rates* using diadochokinetic rate (DDKR) and *irregular sequential motion rates* by diadochokinetic irregularity (DDKI). Using a sustained phonation paradigm to assess phonatory stability, we examined *pitch fluctuations* by the standard deviation of fundamental frequency (F0 SD) and *increased noise* by the harmonics-to-noise ratio (HNR). To examine vocal tremor characteristics, based on the sustained phonation paradigm, we estimated the *vocal vibrato* by modulation depth of frequency tremor (MDFT) and the *vocal tremolo* by modulation depth of amplitude tremor (MDAT). Finally, speech timing characteristics were based on the reading passage paradigm and included an investigation of *prolonged pauses* via duration of pause intervals (DPI) and *slow articulation rate* via net speech rate (NSR). Comprehensive details on feature extraction and validation have been reported previously (Hlavnicka 2018; Hlavnicka et al. 2020); the program code is not publicly accessible. The values of variables were averaged across two repetitions to provide greater speech assessment stability (Rusz et al. 2021a). All analyses were performed in MATLAB^®^ (MathWorks, Natick, MA).

**Table 1:** Overview of applied acoustic speech features

Speech [vocal task]	Objective feature	Definition (unit)	Pathophysiological interpretation
**Oral diadochokinesis**			
Slow sequential motion rates [fast syllable repetition]	DDKR	Diadochokinetic rate, defined as the number of syllable vocalizations per second (syll/s).	Speech apparatus makes the movements of articulators slower.
Irregular sequential motion rates [fast syllable repetition]	DDKI	Diadochokinetic irregularity, defined as the standard deviation of distances between following syllable nuclei (ms).	Inappropriate timing of speech movements.
**Phonatory stability**			
Pitch fluctuations [sustained phonation]	F0 SD	Standard deviation of fundamental frequency contour (semitones).	Uncontrolled alterations in voice pitch.
Increased noise [sustained phonation]	HNR	Harmonics-to-noise ratio, defined as the amplitude of noise relative to tonal components (dB).	Reduced rate of airflow and improper control of vocal folds cause increased turbulent noise.
**Vocal tremor**			
Vocal vibrato [sustained phonation]	MDFT	Modulation depth of frequency tremor, defined as median modulation depth of dominant frequency tremor. The dominant tremor track was determined from the contour of modal fundamental frequency (semitones).	Loss of muscle control leads to involuntary oscillatory movements in the throat, which cause rhythmical or quasirhythmical fluctuations in pitch perceived as quavering of the voice.
Vocal tremolo [sustained phonation]	MDAT	Modulation depth of amplitude tremor, defined as median modulation depth of dominant amplitude tremor. The dominant tremor track was determined from the signal envelope within voiced intervals (%).	Loss of muscle control leads to involuntary oscillatory movements in the throat, which cause rhythmical or quasirhythmical fluctuations in loudness perceived as quavering of the voice.
**Speech timing**			
Prolonged pauses [reading passage]	DPI	Duration of pause intervals, defined as the median length of pause intervals (ms).	Difficult initiation speech and inappropriate timing lead to prolonged pause intervals.
Slow articulation rate [reading passage]	NSR	Net speech rate, defined as the total number of syllables divided by the total duration of speech after removal of pauses (syll/s).	Impaired control of orofacial muscles leads to a decrease in speech rate.

### Statistical analysis

Between-group differences were calculated using an analysis of covariance adjusted by sex and age followed by Fisher’s least-squares difference. A partial Spearman’s correlation adjusted by sex and age was used to assess relationships between speech variables and clinical scales. A two-tailed p-value < 0.05 was considered a threshold for statistically significant differences.

Based on the results of our primary hypothesis, we conducted secondary analysis using a binary logistic regression followed by leave-one-subject-out cross-validation to evaluate the effectiveness of a combination of acoustic features in distinguishing between groups (i.e., accuracy, sensitivity, and specificity). Diagnostic accuracy was assessed using the area under the curve (AUC), derived from the receiver operating characteristic curve. This analysis was performed to indicate potential diagnostic accuracy, and thus was calculated only between CD and ET-HT (i.e., primary hypothesis) and between these groups and controls. Also, there is too small a sample size in the ET-nHT group for classification experiment.

### Data Availability

Individual participant data that underlie the findings of this study are available upon request to the corresponding author by qualified researchers (i.e., affiliated to a respected university or research institution/hospital). The speech data are not publicly available due to their contain of information that could compromise the privacy of study participants.

## Results

### Participants

A total of 93 patients were included in the study (Table 2). Thirty-nine of them (10 male, mean age 62.2 ± 9.0 years) fulfilled the diagnostic criteria of CD, 38 patients (21 male, mean age 65.8 ± 10.7 years) had ET-HT, and 16 patients (10 male, mean age 64.2 ± 17.2 years) had ET-nHT. In addition, 60 volunteers (30 male, mean age 64.1 ± 12.0 years) served as healthy controls for speech testing and statistical adjustment. There were no significant differences in age between groups.

**Table 2:** Clinical characteristics of patients

	CD	ET-HT	ET-nHT	*p*-value
	(*n* = 39, 10 males)	(*n* = 38, 21 males)	(*n* = 16, 10 males)	(ANOVA)
Age (years)	62.2 (9.0, 40–75)	65.8 (10.7, 40–82)	64.2 (17.2, 31–81)	0.4
Age at onset (years)	38.2 (19.0, 6–67)	34.3 (17.1, 4–64)	43.1 (23.0, 6–75)	0.29
TETRAS	17.1 (14.4, 3–59)	38.7 (15.6, 6–74)	22.9 (8.9, 10–47)	< 0.001a, c
TETRAS head tremor	1.77 (0.74, 0–4)	1.53 (1.01, 0–4)	0	< 0.001a, b
TWSTRS	24.5 (12.1, 3–50)	4.5 (7.2, 0–23)	0.1 (0.3, 0–1)	< 0.001a, b
SARA	2.5 (2.6, 0–12)	4.8 (3.2, 0–16)	1.7 (1.6, 0–6)	< 0.001a, c
SARA speech	0.59 (0.91, 0–3)	0.92 (0.97, 0–3)	0.06 (0.25, 0–1)	< 0.01b, c

CD = cervical dystonia, ET + = essential tremor with head tremor, ET- = essential tremor with no head tremor, TETRAS = The Essential Tremor Rating Assessment Scale, TWSTRS = Toronto Western Spasmodic Torticollis Rating Scale, SARA = Scale for the Assessment and Rating of Ataxia, ANOVA = analysis of variance

a Significant difference between CD and ET-HT

b Significant difference between CD and ET-nHT

c Significant difference between ET-HT and ET-nHT

### Speech differences

**Fig. 1:**
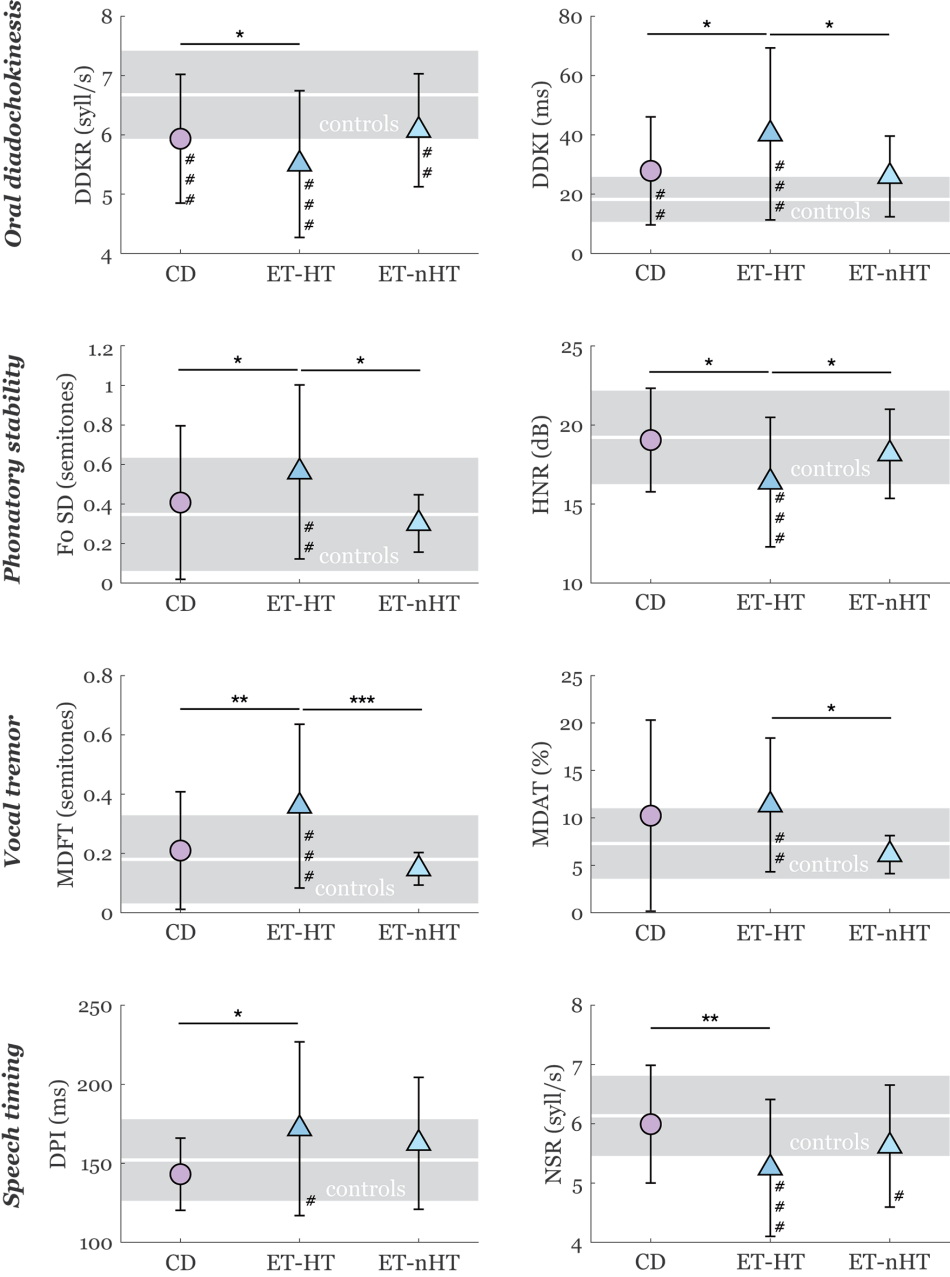
Results of speech analyses. The symbols represent mean values, and error bars represent standard deviation. The shaded area represents the standard deviation for the control group, with a white line in the middle indicating the mean value. Statistically significant differences between CD, ET-HT, and ET-nHT groups after adjustment for sex and age: *p < 0.05, **p < 0.01. Statistically significant differences between patient groups and controls after adjustment for sex and age: #*p* < 0.05, ##*p* < 0.01, ###*p* < 0.001. CD = cervical dystonia, ET-HT = essential tremor with head tremor, ET-nHT = essential tremor with no head tremor, DDKR = diadochokinetic rate, DDKI = diadochokinetic irregularity, F0 SD = standard deviation of fundamental frequency, HNR = harmonics-to-noise ratio, MDFT = modulation depth of frequency tremor, MDAT = modulation depth of amplitude tremor, DPI = duration of pause intervals, NSR = net speech rate

Compared to CD, ET-HT showed slower and more irregular sequential motion rate (DDKR: *p* = 0.04; DDKI: *p* = 0.03), excessive pitch fluctuations (F0 SD: *p* = 0.04), increased noise (HNR: *p* = 0.01), higher vocal vibrato (MDFT: *p* = 0.002), prolonged pauses (DPI: *p* = 0.01) and slower articulation rate (NSR: *p* = 0.002) (Fig. 1). Similar results were found when comparing ET-HT to ET-nHT, with ET-HT having greater irregular sequential motion rates (DDKI: *p* = 0.02), pitch fluctuations (F0 SD: *p* = 0.02), increased noise (HNR: *p* = 0.049), and extent of both vocal vibrato and tremolo (MDFT: *p* < 0.001; MDAT: *p* = 0.02). No significant differences were found between CD and ET-nHT groups, although both ET groups had slower articulation rate than controls while it was intact in CD. No speech differences were observed between male and female controls (Table S1).

Considering abnormal vocal tremor on individual patient level using previously estimated cut-off values of 0.5 semitones (frequency) and 20% (amplitude) (Hlavnicka et al. 2020), 3 CD patients exhibited abnormal vocal vibrato (mean 4.4, SD 0.2, range 4.2–4.6 Hz) and 4 CD patients vocal tremolo (mean 4.0, SD 1.5, range 1.8–5.2 Hz), while abnormal vocal vibrato was found in 11 ET-HT patients (mean 3.5, SD 1.3, range 1.2–5.8 Hz) and vocal tremolo in 4 ET-HT patients (mean 4.4, SD 0.9, range 3.6–5.5 Hz) (Video S1). No ET-nHT patient showed abnormal vocal tremor.

The only weak correlation was observed in the entire patient sample between head tremor and vocal tremolo (TETRAS Performance Scale item 1 and MDAT: *r* = 0.25, *p* = 0.01).

### Sensitivity analysis

**Fig. 2:**
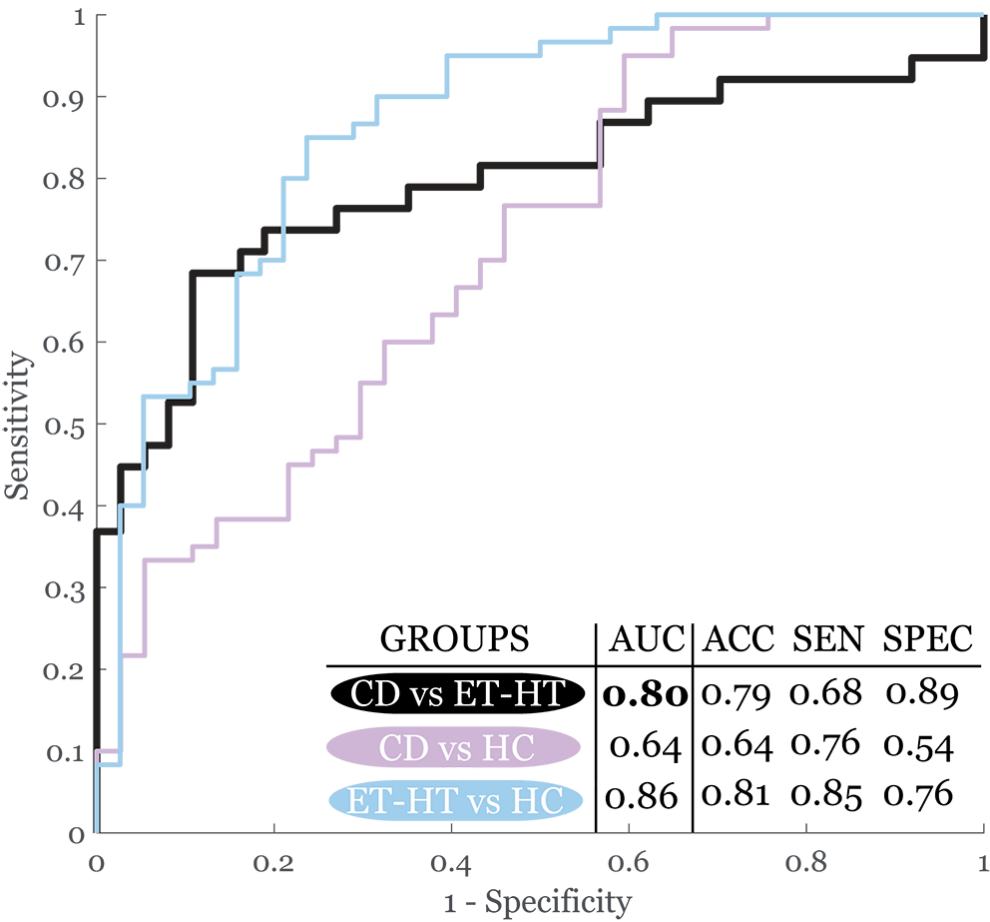
Receiver operating characteristic curves with the listed accuracy. AUC = area under curve, ACC = accuracy, SEN = sensitivity, SPEC = specificity, CD = cervical dystonia, ET-HT = essential tremor with head tremor, HC = healthy controls

The combination of 5 features representing irregular sequential motion rate (DDKI), increased noise (HNR), vocal vibrato and tremolo (MDFT, MDAT), and prolonged pauses (DPI) led to the best overall discrimination between CD and ET-HT groups with AUC of 0.80 (Fig. 2).

## Discussion

The differential diagnosis of essential and dystonic head tremor can be challenging, especially in patients where head tremor predominates (Defazio et al. 2015; Elble 2013; Lalli and Albanese 2010;. Pandey et al. 2020). This study is the first to show that assessing speech abnormalities can contribute to this diagnostic challenge. Indeed, we could differentiate between CD and ET-HT groups with a good AUC of 0.80. But the question goes further, touching on the pathophysiological mechanisms of tremor. Speech analysis can be of special interest here, discerning individual features of voice and articulation reflecting e.g. dystonic spreading of muscle activation or disordered cerebellar role in timing of movements.

While previous studies have already demonstrated phonatory control issues in ET patients (Gamboa et al. 1998; Hlavnicka et al. 2020; Suppa et al. 2021), our results showed that the impairments including pitch fluctuations and increased noise primarily affect individuals with ET-HT. These are typically associated with improper control of vocal folds and are encountered in most neurological diseases manifesting hyperkinetic dysarthria (Rusz et al. 2021a). ET-HT also showed a higher extent of vocal tremor with approximately 4 Hz frequency, which is in line with previous literature on vocal tremor in ET (Sulica and Louis 2010), as well as with presumed pathomechanisms reflecting basal ganglia dysfunction (Cagnan et al. 2014). Interestingly, these changes were predominantly observed in ET-HT patients, although some CD patients also exhibited abnormal vocal tremor, while no such tremor was found in ET-nHT patients, suggesting a stronger association with the presence of head tremor. The correlation found between acoustic vocal tremor and clinical measurement of head tremor in the TETRAS scale can further support such an assumption.

More intriguingly, we found speech timing abnormalities, including slow and irregular sequential motion rates and prolonged pauses, particularly in CD and ET-HT groups, although these speech timing issues were significantly more severe in ET-HT. We might hypothesize that these findings are related to cerebellar involvement (Schalling et al. 2007), which has been widely documented in ET (Louis 2018) but also in CD (Kaji et al. 2018). Indeed, due to sequential planning and programming deficits, diadochokinesis irregularity has been assumed to be the most characteristic of ataxic dysarthria (Ackermann and Hertrich 2000; Ziegler and Wessel 1996). A previous study on volumetric correlates found that slow oral diadochokinesis in multiple sclerosis is particularly related to the extent of cerebellar atrophy (Rusz et al. 2019). In addition, only ET groups manifested a slow speaking rate, which is another speech dysfunction frequently appearing in cerebellar ataxia (van Prooije et al. 2024).

In general, the extent to which voice abnormalities in dystonia overlap with or differ from those in ET remains unclear, limiting a comprehensive understanding of the pathophysiological mechanisms underlying the diverse manifestations of voice tremor. Despite that our CD cohort had significantly altered only oral diadochokinesis, previous studies frequently observed a hyperkinetic dysarthric profile in patients with dystonia, characterized by imprecise consonants, a rough voice, changes in intensity, and hypernasality (Barkmeier-Kramer and Clark 2017; Cuartero et al. 2021; Kreisler et al. 2016), suggesting that articulatory structures (tongue, lips, and jaw), as well as laryngeal tremor, may be involved as comorbid regions affecting speech. To the best of our knowledge, the only study on neural representation of the voice tremor spectrum has found a broad overlap between cortical alterations in essential voice tremor and dystonic voice tremor, yet there were also unique patterns of abnormalities in regions controlling speech motor preparation, which were localized in the cerebellum in essential voice tremor and the premotor cortex, insula, and superior temporal gyrus in dystonic voice tremor (de Lima Xavier and Simonyan 2020). This finding aligns with our observation that the ET-HT group exhibited the most pronounced cerebellar abnormalities.

One potential limitation is that we were not able to match CD and ET-HT groups in sex, as dystonia is known to have a higher prevalence in females (Kilic-Berkmen et al. 2024) and ET in males (Song et al. 2021).^39^ On the other hand, all statistical analyses were adjusted by sex, and we also performed subgroup analysis for controls confirming that sex did not significantly affect the investigated speech features. We observed a weak to no correlation between speech and clinical scales, which might imply that speech dysfunction represents non-overlapping/complementary marker of disease severity. However, future research should consider comparing speech dysfunction with a more detailed instrumental analysis of motor symptoms for greater insight. Our findings are based on fully automated acoustic analyses, which require further validation using independent cohorts and complementary methodologies. For example, previous research has shown that endoscopic voice evaluation can be particularly helpful in distinguishing between essential and dystonic voice tremor (Moraes and Biase 2016).

## Conclusion

The present study demonstrates a promising advance in speech analysis that may help distinguish the origin of head tremor in patients with uncertain diagnoses. It confirms the greater severity and distinct pattern of speech impairment in ET-HT patients compared to those with CD. Our results support the critical role of cerebellar control circuits involvement in the speech of patients with both CD and ET. Automated acoustic analysis offers a cost-effective, non-invasive method for potential screening, requiring no advanced technical equipment and being adaptable across different languages (Rusz et al. 2021b). Future multicentric studies are needed to confirm the present findings and collect a larger sample size, allowing predictive models with higher statistical power to be built.

## Electronic supplementary material

Below is the link to the electronic supplementary material.

Supplementary material 1

Supplementary material 2
