# Supplementary material for: Unravelling head tremor mechanisms: insights from speech analysis in essential tremor and cervical dystonia
Source: J Neural Transm (Vienna). 2025 Jun 7;132(8):1171–8. doi: 10.1007/s00702-025-02965-5 (PMC12479614; doi:10.1007/s00702-025-02965-5)
Supplement: Supplementary file 1 — Supplementary material 1 [file 702_2025_2965_MOESM1_ESM.docx]

**Table S1.** Results of comparison between male and female healthy speakers.

|  | Female | Male | p-value (t-test) |
| --- | --- | --- | --- |
| **Oral diadochokinesis** |  |  |  |
| DDKR (syll/s) | 5.9 (1.2, 2.2-8.6) | 5.7 (1.1, 3.0-8.6) | 0.45 |
| DDKI (ms) | 31.5 (16.8, 9.3-79.9) | 31.1 (24.4, 9.9-138.1) | 0.95 |
| **Phonatory stability** |  |  |  |
| F0 SD (semitones) | 0.49 (0.44, 0.15-1.88) | 0.47 (0.39, 0.16-1.64) | 0.88 |
| HNR (dB) | 18.3 (3.7, 9.6-24.4) | 17.4 (3.7, 9.9-24.3) | 0.32 |
| **Vocal tremor** |  |  |  |
| MDFT (semitones) | 0.28 (0.22, 0.08-0.90) | 0.27 (0.26, 0.08-1.14) | 0.97 |
| MDAT (%) | 11.7 (11.8, 2.6-62.8) | 9.9 (7.7, 3.1-31.4) | 0.48 |
| **Speech timing** |  |  |  |
| DPI (ms) | 146 (30, 87-245) | 154 (29, 103-243) | 0.28 |
| NSR (syll/s) | 5.8 (1.0, 3.4-8.1) | 5.8 (1.1, 3.9-8.8) | 0.97 |
| Data are presented as mean (SD, range). | |  |  |
| DDKR = diadochokinetic rate, DDKI = diadochokinetic irregularity, | | |  |
| F0 SD = standard deviation of fundamental frequency, HNR = harmonics-to-noise | | | |
| ratio, MDFT = modulation depth of frequency tremor, MDAT = modulation depth | | | |
| of amplitude tremor, DPI = duration of pause intervals, NSR = net speech rate. | | | |
